# Supplementary material for: The Development and Validation of a Satisfaction and Emotional Perception Scale for Women Undergoing Fertility Treatment
Source: Healthcare (Basel). 2025 Sep 24;13(19):2416. doi: 10.3390/healthcare13192416 (PMC12523600; doi:10.3390/healthcare13192416)
Supplement: Supplementary file 1 [file healthcare-13-02416-s001.zip › healthcare-3762957-supplementary.pdf]

Supplementary Materials

Please rate from 1 to 10 what you consider:

Item 1 “I feel sad and depressed because of my reproductive problems”.

0	1	2	3	4	5	6	7	8	9	10

Item 2 "I am satisfied with the support I receive from my family and/or friends about my reproductive problems".

0	1	2	3	4	5	6	7	8	9	10

Item 3 "I am satisfied with my sexual relations, even if I have reproductive problems".

0	1	2	3	4	5	6	7	8	9	10

Item 4 "My reproductive problems have caused negative repercussions in my relationship".

0	1	2	3	4	5	6	7	8	9	10

Item 5 “Reproductive treatment has a negative effect on my mood”.

0	1	2	3	4	5	6	7	8	9	10

Item 6 “I have the care of the Assisted Human Reproduction Unit (AHRU) nurse I want”.

0	1	2	3	4	5	6	7	8	9	10

Item 7 "I feel that the nurse in charge of my treatment understands what I am going through".

0	1	2	3	4	5	6	7	8	9	10

Item 8 " I am satisfied with the quality of the services available to me to meet my emotional needs”.

0	1	2	3	4	5	6	7	8	9	10

Item 9 " The information I received about the medicines I am taking or have taken and/or treatments I am receiving or have received was adequate".

0	1	2	3	4	5	6	7	8	9	10

Item 10 "I feel that the emotional support provided by the nurse was sufficient to reduce my level of anxiety".

0	1	2	3	4	5	6	7	8	9	10

Item 11 "After receiving the information provided by the nurse during the process, my level of anxiety about the reproductive treatments has decreased".

0	1	2	3	4	5	6	7	8	9	10
